# Exploring associations between household environmental factors and handwashing with essential agents in sub-Saharan Africa

**DOI:** 10.1371/journal.pone.0286735

**Published:** 2023-06-29

**Authors:** Aiggan Tamene, Aklilu Habte, Mihretu Tagesse, Fitsum Endale, Tamirat Melis, Zablon Wale Sewalem, Abel Afework

**Affiliations:** 1 School of Public Health, College of Medicine and Health Sciences, Wachemo University, Hosaena, Ethiopia; 2 Department of Public Health, College of Medicine and Health Sciences, Wolkite University, Wolkite, Ethiopia; 3 Department of Clinical and Psychosocial Epidemiology, University of Groningen, Groningen, the Netherlands; 4 Department of Environmental Health, College of Medicine and Health Sciences, Dilla University, Dilla, Ethiopia; University of KwaZulu-Natal College of Health Sciences, SOUTH AFRICA

## Abstract

**Background:**

3 billion people lack proper home hand hygiene facilities globally. Of these, 1.4 billion (18%) lack soap or water, while 1.6 billion (22%) have neither. This analysis explores the link between living conditions and the use of essential agents in sub-Saharan Africa. This secondary data analysis examines potential associations between the domiciliary environment and the use of essential agents in sub-Saharan Africa.

**Methods:**

Eighteen demographic and health surveys were used to analyze the association between household environmental factors and handwashing with essential agents. STATA version 16 was used to analyze data from 203,311 households across weighted samples. Using a multivariable multilevel mixed effect logistic regression analysis, it was possible to determine how each independent factor affected the outcome while taking the data clustering into account. The adjusted odds ratio and its associated 95% confidence interval were used to assess the independent factors’ statistical significance.

**Result:**

Only one in three households 34.84%, practiced handwashing with essential agents, with the highest prevalence in Angola (70.2%) and the lowest in Malawi (6.5%). Educational status [aOR = 1.77; 95%(CI = 1.68–1.86)], female headship[aOR = 1.09; 95%(CI = 1.06–1.2)], household wealth[aOR = 4.08; 95%(CI = 3.84–4.33)], not sharing toilets with other homes[aOR = 1.13; 95%(CI = 1.10–1.17)], having a fixed place for hand washing[aOR = 1.49; 95%(CI = 1.45–1.54)], not having regular access to water [aOR = 0.09; 95%(CI = 0.095–0.10)]and being a rural resident [aOR = 0.85; 95%(CI = 0.82–0.88)] were associated with handwashing.

**Conclusion:**

sub-Saharan nations are failing to demonstrate advancements in handwashing practices. There are still a lot of homes without access to basic infrastructure for handwashing and household water sources. For essential agent adoption programs to be successful in an environment with limited resources, Water, Sanitation, and Hygiene measures must be implemented. Furthermore, it is critical to include contextual factors from the current study as well as socio-cultural and psychological characteristics that dissuade people from using essential agents in intervention strategies.

## Introduction

Handwashing is the act of cleaning hands for 10–15 seconds by using essential agents [1]. An important indicator for the global monitoring of hygiene is the availability of a handwashing station with the necessary components (water and soap) on the premises. A handwashing facility is defined as a device that "may be fixed or mobile and include a sink with tap water, buckets with taps, tippy-taps, and jugs or basins dedicated for handwashing" by the World Health Organization (WHO) and the United Nations Children’s Fund (UNICEF). Ash, soil, sand, and other handwashing materials are not included in the definition of soap, which also includes liquid soap, powder detergent, and soapy water [2, 3].

The WHO advises washing hands frequently, but especially before and after caring for anyone who may be ill; before, during, and after preparing food; before eating; after using the restroom; after assisting someone who has just done so; after blowing one’s nose; after coughing or sneezing; after touching an animal, animal feed, or animal waste; and after touching garbage [4].

Regular handwashing has a significant impact on one’s capacity to maintain good health. Maintaining hand cleanliness promotes good nutrition, improves educational possibilities by reducing absences from class, and prevents disease, which promotes child development and, in turn, increases economic potential [5, 6]. Handwashing with essential agents may reduce diarrhea by up to 42%, pneumonia, and other respiratory diseases by a third, and parasite infections by half, according to evidence from randomized controlled trials [7–9]. The recent pandemic brought on by the SARS-CoV-2 serves as more evidence of the value of handwashing with essential agents in reducing the risk of infection [10, 11].

However, developing the practice of consistent handwashing remains difficult, particularly in developing world contexts where it has been noted that only 3–35% of people wash their hands at crucial times [12, 13]. In the least developed nations, over three out of every four residents lack access to essential agents at home. In 2017, the WHO and UNICEF reported that 3 billion people globally lacked access to adequate home hand hygiene facilities (HHFs): 1.6 billion (22%) had HHFs that were lacking in terms of soap or water, and 1.4 billion (18%) had none whatsoever [14].

The current state of knowledge on the household factors associated with handwashing with essential agents in sub-Saharan Africa is still evolving. Recent studies have highlighted several important factors that influence handwashing practices in households, including access to water and sanitation facilities, education and awareness, socio-economic status, and cultural beliefs and practices [15–18]. However, a more comprehensive understanding of the diverse and nuanced factors that drive handwashing behavior in various communities and settings across the region is still necessary. To achieve this, it is important to conduct further research that adheres to the latest guidelines from public health organizations and local authorities. This knowledge can be used to develop effective interventions to promote handwashing with essential agents and ultimately improve public health outcomes in sub-Saharan Africa.

The scant literature on the multilevel factors that determine the practice of handwashing with essential agents reveals that we still lack scalable models for hygiene and sanitation that can ensure desired practices in circumstances where risks are common, compliance costs are high, and enforcement capacity is weak. This study thus aimed to examine the fixed effects (measures of association) and the random effects (measures of variation) of handwashing behavior with essential agents in 18 countries in sub-Saharan Africa.

## Methods

### Study design and area

Cross-sectional data from 18 nationally representative Demographic and Health Surveys conducted between 2016 and 2021 are used in this report. The Demographic and Health Survey(DHS) Program offers extensive secondary data collected through surveys using probability sampling techniques and adhering to recognized international standards [19]. The survey makes use of multiple sets of questionnaires that have been developed and pretested to ensure reliability and the capacity to compare data acquired on various spatial and temporal scales [20]. As data collection techniques have become more reliable over time, only the most recent country survey was used in this research to ensure comparability of results for individual countries and to avoid giving extra weight to nations that have several surveys in the cumulative estimates.

### Study countries

A total of 203,311 households were included in the study, which drew a weighted sample from 18 countries in sub-Saharan Africa across four regions, namely Western, Eastern, Central, and Southern Africa (refer to Table 1). Prior to country selection, certain criteria were established: the country had to be situated in sub-Saharan Africa according to the United Nations regional classifications, and a demographic and health survey dataset with standardized questions and observations on handwashing as well as other relevant domiciliary factors had to be available post-2016. Notably, the Demographic and Health Survey(DHS) survey post-2016 excluded materials such as ash, soil, sand, and other handwashing substances from the definition of soap, following the guidelines of the World Health Organization, and instead encompassed liquid soap, powder detergent, soapy water, and disinfectant wipes as cleaning agents.

**Table 1:** Description of the sub-Saharan African countries included in the analysis, 2016–2021.

Regions	DHS Year	Weighted sample size
**Central Region**	2016–2018	
Angola	2016	6,099
Cameroon	2018	11,129
**Eastern Region**	**2016–2021**	
Burundi	2017	15,757
Ethiopia	2016	9,966
Madagascar	2021	17,826
Malawi	2017	21,977
Rwanda	2020	10,831
Uganda	2016	11,592
Zambia	2018	6,824
Mauritania	2020	8,956
**Western Region**	**2018–2020**	
Benin	2018	7,797
The Gambia	2020	5,832
Guinea	2018	5,659
Liberia	2021	1,883
Mali	2018	6,773
Nigeria	2018	32,696
Sierra Leone	2020	5,474
**Southern region**	**2016**	
South Africa	2016	9,467
**Total**	**2016–2021**	203, 311

### Data source and study period

The Demography Health Survey (DHS) program’s official database, www.measuredhs.com was accessed for the sake of this investigation after permission was acquired via an online form that outlined the study’s objectives.

### Study variables

#### Outcome variable

The dependent variable for this study was the prevalence of "handwashing practice with essential agents in households." During interviews conducted by the Demographic and Health Survey, participants were requested to show the area within their household where handwashing was predominantly performed. This was done to gather data on variables related to handwashing responses. The sources of water used for handwashing were classified as either unimproved or improved. Furthermore, households were asked about the availability of hand cleaning agents, with responses ranging from soap and detergent to ash, mud, and sand. The availability of the two fundamental handwashing supplies, soap, and improved water, was examined in homes.

The dependent variable was then split into two categories: "Yes" for households that had engaged in necessary handwashing practices during the study period and "No" for households that had no [5].

#### Independent variables

After reviewing recent literature, potential independent predictors of essential agent use were taken from the data set. Household-level variables were defined as respondent variables that were unique to each household [5, 6, 12, 13](Table 2).

**Table 2:** Household-level variables extracted from the demographic and health survey 2016–2021 data set for studying factors associated with essential agent use.

Variable	Description	Category
**Age of household (HH) head**	The respondent’s age, expressed in years, at the time of the survey.	1. <18 2. 19–30 3. 31–45 4. 46–65 5. >66
**Educational Status of HH head**	The respondent’s highest degree of education at the time of the survey	0. No formal education 1. Primary education 2. Secondary education 3. higher education
**Household wealth**	Households received ratings based on the quantity and kind of consumer goods they owned.	0. Poorest 1. Poor 2. Middle 3. Rich 4. Richest
**Family Size**	The number of people who lived in the house	0. <3 1. 4–7 2. >7
**Toilet type [18]**	It is separated into two categories: improved (any non-shared flush/pour flush toilet of the following types: ventilated improved pit [VIP] latrines, pit latrines with slabs, and composting toilets); and unimproved (shared toilet, flush/pour flush not to sewer/septic tank/pit latrine, pit latrine without slab/open pit, hanging latrine, and others).	0. Improved 1. Unimproved
**Water source [21]**	Either listed as unimproved sources (like an unprotected dug well and spring, a tanker truck or cart with a small tank, and surface water) or improved sources (such as piped water, public taps, standpipes, tube wells, boreholes, protected dug wells and springs, and bottled water).	0. Improved 1. Unimproved
**Location of water source**	The location of the drinking water source	0. On-Premises 1. Off-premises
**Share toilets**	Whether toilets are shared between a group of households in a single building or plot.	0.No 1.Yes
**Time to collect water**	How long it takes to get water	0. <30minutes 1. >30minutes
**Treat water [22]**	As far as water treatment methods go, boiling, adding bleach, filtering, and solar disinfection (SODIS) are all considered to be efficient.	0.No 1.Yes
**Service continuity**	The past 14 days have seen at least one full day’s worth of water service interruptions.	0.No 1.Yes

The factors applied at the community level were those that affected every home within the same community (cluster), such as residence, sub-region, community (cluster) poverty, and community education (Table 3). The cluster’s components were combined to yield variables like community poverty and education levels.

**Table 3:** Community- level variables extracted from the demographic and health survey 2016–2021 data set for studying factors associated with essential agent use.

Variable	Description	Category
**Place of residence**	The area where respondents lived when the survey was conducted.	0. Urban 1. Rural
**sub-Region [23]**	Central or Middle African countries—Angola, Cameroon, Southern African countries—South Africa East African countries- Burundi, Ethiopia, Kenya, Madagascar, Malawi,Rwanda, Tanzania, Uganda, Zambia, and Zimbabwe Western Africa- Benin, Gambia, Guinea, Liberia, Mali, Mauritania,Nigeria, Sierra Leone	1.Central Africa 2.East Africa 3.West Africa 4.South Africa
**Community Poverty**	Described as the percentage of respondents who lived in the cluster’s poorest homes. The cluster’s overall poverty can be determined by adding up the individual households with the lowest wealth indices.	0. Low 1. High
**Community Education**	Described as the percentage of respondents in the cluster who attended primary, secondary, or higher education. The sum of each respondent’s primary, secondary, and higher education levels might reveal the cluster’s overall educational achievement.	0. Low 1. High

### Data management and statistical analysis

Using STATA version 14, variables were organized, cleaned, and recoded before analysis. Households that had missing or unavailable outcome variables—those whose values couldn’t be used—were not included. These data were entered into the database using a unique code called "na," which either denoted "Don’t know" or replies that were deemed to be inconsistent with other entries on the questionnaires and hence likely to be erroneous.

#### Multilevel analysis

First, frequencies and proportions were computed using analytical and descriptive statistics. A weighted analysis was conducted to take into consideration the unequal likelihood of selection between the strata brought on by the non-proportional distribution of samples to various sub-regions, residences, and non-response rates.

Due to the Demographic and Health Survey data’s hierarchical structure, where individuals are nested within households and households are nested inside clusters, multilevel (two-level) regression was used. The mixed-effect multilevel regression (MEMR) was the preferred statistical technique because of its exceptional flexibility and power in analyzing complex data that has a hierarchical structure, such as clustered data [24]. This method enables the modeling of the relationships between predictor variables and a response variable while taking into account dependencies within-subjects or clusters. MEMR is highly adept at handling intricate data structures, including those with multiple levels of nesting, such as observations nested within districts. Additionally, it can manage missing data and unbalanced designs, making it a versatile and robust technique [25].

Following the bi-variable multilevel logistic regression analysis, the variables with a p-value less than 0.25 were included in the multivariable analysis. To demonstrate the strength of the association, an adjusted odds ratio with a corresponding 95% confidence level was produced. For independent factors having a p-value of 0.05 or below, it was determined that the dependent variable was significantly associated with them. Multicollinearity between the household and community-level variables was examined using the Variance Inflation Factor (VIF) <10.

#### Random effects

Four random intercept models were fitted (Models 1, 2, 3, and 4).

Model 1 (null model): This model is based solely on the intercept and does not include any other independent variables. Statistics for measures of variance (random effects) were computed using the intra-class correlation coefficient (ICC) and median odds ratio (MOR). MOR can quantify unexplained cluster variability, whereas ICC explains cluster variability (heterogeneity) [26, 27]. The ICC was 24%, demonstrating that variations at the cluster level were what caused variations in the prevalence of handwashing with essential agents. An ICC of at least 2%, which is needed for a multilevel study design, indicates significant group-level variance. Variables at the household and community levels have been considered in Models 2 and 3, respectively.

Household and community-level factors were both taken into consideration in Model 4. This model indicated that 11.7% of the unexplained variation might be explained by unobserved community- and individual-level factors. All models were compared and the fourth model with the lowest deviance was selected as the best-fit model. Additionally, were used to evaluate the goodness of fit. Better explanatory models are considered to have lowered AIC or BIC values [28] (Table 4).

**Table 4:** Random intercept variances and model fit statistics comparison of two-level mixed effect logistic regression model.

Measures	Null Model (Model 1)	Model-II	Model-III	Model-IV
**Random effects**				
ICC	0.24	0.12	0.19	0.117
PCV	Ref	0.545	0.250	0.61
MOR	2.67	1.94	2.34	1.85
AIC	243938	156367	229661.2	153451
BIC	243959	156617	229742.9	153763
**Model fitness**				
Deviance	234934.64	156317.012	229645.2	152834.406

### Ethics approval and consent to participate

All methods and procedures were carried out per the relevant guidelines and regulations of the Declaration of Helsinki. The Demographic and Health Surveys (DHS) collected written informed consent from all subjects and/or their legal guardian during the primary data collection. The retrieved data were only used for the registered research, and data were not shared with anyone other than the co-authors. Data were also fully anonymized by the Demographic and Health Survey program before the author team accessed them. No attempt was made to identify any household or individual, and the information was kept confidential. All data collected by the Demographic and Health Survey (DHS) are Institutional Review Board (IRB) certified and this secondary analysis received further IRB approval from the Wachemo University College of Medicine and Health Science Research Determination Committee.

## Result

### Socio-demographic characteristics

The weighted sample for this study included 203, 311 households. 36.67% of the study participants were household heads between the ages of 31 and 45. Likewise, 50.7% of the homes had four to seven people in residence. In the present study, 32% of the household heads had no formal education. 26.56% of the homes were female-headed households. 35.8% of households fell into the two quintiles with the lowest levels of wealth. In terms of the participants’ characteristics at the community level, 62.59% of the participants were rural residents. In addition, East Africa was home to 46.91% of the households(Table 5).

**Table 5:** Distribution of household and community-level socio-demographic factors, analysis from 18 countries, 2016–2021.

Variable	Frequency	Percentage
**Age of household head**		
<18	1,167	0.57
19–30	40,703	20.02
31–45	74,556	36.67
46–65	63,954	31.46
>66	22,927	11.28
**Educational status of HH head**		
No formal education	66,299	32.61
Primary education	65,639	32.28
Secondary education	50,381	24.78
Higher education*	20,992	10.32
**Sex of HH head**		
Male	149.305	73.44
Female	54,006	26.56
**Household wealth**		
Poorest	35,066	17.25
Poorer	37,717	18.55
Middle	39,705	19.53
Richer	42,728	21.02
Richest	48,095	23.66
**Family Size**		
<3	69,077	33.98
4–7	102,870	50.60
>7	31,364	15.43
**Place of residence**		
Urban	76,051	37.41
Rural	127,259	62.59
**Sub-Region**		
West Africa	81,844	40.26
Middle Africa	17,228	8.47
Eastern Africa	94,772	46.91
Southern Africa	9,447	4.66
**Community Poverty**		
Low	104,332	51.32
High	98,979	48.68
**Community Education**		
Low	102,966	50.64
High	100,345	49.36

*College degree, Vocational degree

HH- household

### Water, sanitation, and hygiene related characteristics of households

Only 49.36% of the homes had access to improved sanitation, while 58.04% had access to improved water sources. 67.74% of households accessed water from places other than their residences. In 37.1% of the studied households, there were shared toilets. In 16.6% of the houses, the distance to the water source was greater than 30 minutes. Mobile handwashing stations were available in 70% of the homes. Comparably, only over a quarter of the households (24.49%) treated their water at the point of use before utilizing it. In the 14 days before the study, water service interruption was reported in 57.20% of the homes as lasting a full day or longer. In the handwashing stations, only half of the examined households had running water(Table 6).

**Table 6:** Distribution of water, sanitation, and hygiene-related factors, analysis from 18 countries, 2016–2021.

Variable	Frequency	Percentage
**Type of toilet facility**		
Unimproved	87,101	50.64
Improved	100,345	49.36
**Water source**		
Improved	118,001	58.04
Unimproved	85,310	41.96
**Location of water source**		
On-premises	65,556	32.25
Off Premises	137,745	67.75
**Share toilet with other home/s**		
Yes	64,207	37.10
No	108,842	62.90
**Water collection time**		
<30minutes	169,569	83.40
>30 minutes	33,742	16.60
**The place where members wash their hands**		
Fixed place	59,126	29.08
Mobile place	144,185	70.92
**Effective water treatment**		
Yes	49,793	24.49
No	153,518	75.51
**Water service continuity (last 14 days)**		
Yes, Interrupted for a full day or more	116,292	57.20
No, not interrupted for a full day	87,018	42.80
**Presence of water at hand washing stations**		
Water not available	101,384	49.87
Water is available	101,927	50.13

### Handwashing practice with essential agents

**Fig 1:**
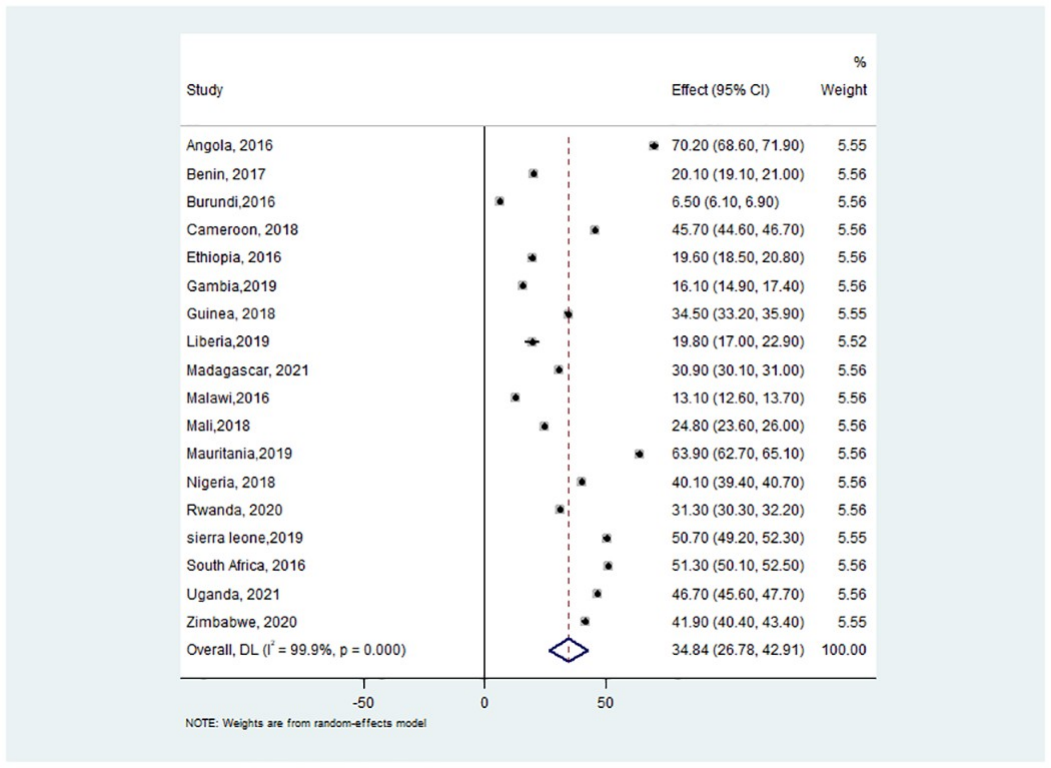
Forest plot depicting pooled prevalence estimate of hand washing practice with essential agents among households in 18 countries in sub-Saharan Africa, 2016–2021.

Interviewers were asked to inspect the location where family members wash their hands most frequently to collect information for the survey on handwashing. At the handwashing stations, soap and water were seen to be present. The dependent variable was then split into two categories: "Yes" for homes with handwashing stations equipped with essential agents and "No" for those without. Only 34.84% of the assessed homes had access to necessary amenities where family members could wash their hands (Fig 1).

### Subgroup analysis

**Fig 2:**
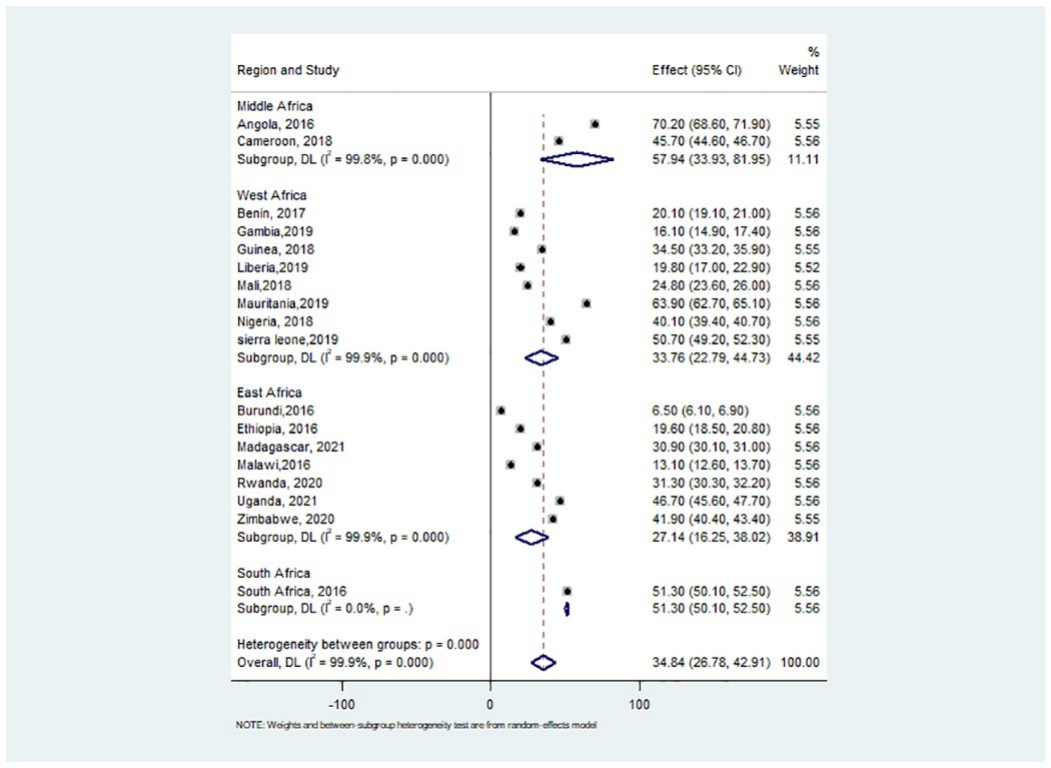
Sub-group pooled prevalence estimate of hand washing practice with essential agents by sub-region among households in 18 countries in sub-Saharan Africa, 2016–2021.

We performed a subgroup analysis based on the region of the survey in this analysis. As a result, the Eastern Africa and Central Africa regions had the highest and lowest prevalence of handwashing with essential agents respectively (Fig 2).

#### Factors associated with handwashing practice with essential agents: A bivariate regression analysis

Table 7 illustrates the unadjusted or crude odds ratio (COR) values that were obtained when the analysis only took into account the impact of one independent variable. Accordingly, the bivariate multilevel logistic regression posted associations between age, respondents’ educational level, wealth index, sex of the household head, and family size and hand washing with essential agents (Table 7).

**Table 7:** Multilevel bivariate logistic regression analysis of factors associated with handwashing with essential agents, analysis from 18 countries, 2016–2021.

Variable categories	Hand washing with essential agents		COR (95% CI)	p-value
	No	Yes		
**Age of household head**				
<18	886 (0.65)	331 (0.50)	1	
19–30	27,869(20.50)	11,856 (18.0)	1.15 (1.00–1.31)	0.038
31–45	48,414 (35.61)	24,049 (36.61)	1.32 (1.15–1.50)	< 0.0001
46–65	42,334 (31.14)	22,139 (33.70)	1.40 (1.23–1.60)	< 0.0001
>66	16,443(12.1)	7,319 (11.14)	1.18 (1.04–1.35)	0.011
**Educational status of HH head**				
No formal education	52,209 (38.4)	14,282 (21.74)	1	
Primary education	47,055 (34.61)	18,281 (27.83)	1.36 (1.33–1.40)	< 0.0001
Secondary education	28,230 (20.77)	21,288 (32.40)	2.56 (2.49–2.63)	< 0.0001
Higher education	8,452 (6.22)	11,843 (18.03)	4.76 (4.60–4.93)	< 0.0001
**Sex of HH head**				
Male	99,498(73.19)	47,154 (71.78)	1	
Female	36,448 (26.81)	18,540 (28.22)	1.06 (1.04–1.09)	< 0.0001
**Household wealth**				
Poorest	31,442 (23.13)	5,152 (7.84)	1	
Poorer	29,326 (21.57)	8.454 (12.87)	1.7 (1.63–1.77)	< 0.0001
Middle	28,702 (21.11)	11,250 (17.12)	2.27 (2.19–2.36)	< 0.0001
Richer	25,668 (18.88)	15,242 (23.2)	3.37(3.25–3.50)	< 0.0001
Richest	20,808 (15.31)	25,596 (38.96)	7.18 (6.93–7.45)	< 0.0001
**Family Size**				
<3	45,099 (33.17)	24,058 (36.62)	1.11 (1.08–1.14)	< 0.0001
4–7	69,066 (50.80)	32,181 (48.99)	1.02 (0.99–1.05)	0.053
>7	21,781 (16.02)	9,454 (14.39)	1	

Similar to this, the bivariate multilevel logistic regression established an association between hand washing with essential agents and the type of toilet facility, type of water source, location of the water source, sharing a toilet with another home or homes, water collection time, the place where members wash their hands, and water treatment (Table 8).

**Table 8:** Multilevel bivariate logistic regression analysis of factors associated with handwashing with essential agents, analysis from 18 countries, 2016–2021.

Variable categories	Hand washing with essential agents		COR (95% CI)	p-value
	No	Yes		
**Type of toilet facility**				
Unimproved	67,440 (49.61)	20,447 (31.12)	1	
Improved	68,506 (50.39)	45,247 (68.88)	2.08 (2.04–2.13)	< 0.0001
**Water source**				
Improved	74,133 (54.53)	43,549 (66.29)	1	
Unimproved	61,813 (45.47)	22,145 (33.71)	0.6 (0.56–0.61)	< 0.0001
**Location of water source**				
On-premises	34,200 (25.16)	30,744 (46.80)	2.63 (2.57–2.68)	< 0.0001
Off Premises	101, 746 (74.84)	34,950 (53.20)	1	
**Share toilet with other home/s**				
Yes	67,864 (61.96)	39,060 (66.04)	1	
No	41,662 (38.04)	20,082 (33.96)	1.24 (1.21–1.27)	<0.0001
**Water collection time**				
<30minutes	110,249 (81.0)	57,768 (87.93)	1.60 (1.55–1.65)	< 0.0001
>30 minutes	25,697 (18.9)	7,926 (12.07)	1	
**The place where members wash their hands**				
Fixed place	28,759 (21.15)	29,889 (45.51)	3.18 (3.12–3.25)	<0.0001
Mobile place	107,187 (78.85)	35,795 (54.49)	1	
**Effective water treatment**				
Yes	28,430 (20.91)	19,352 (29.46)	0.58 (0.56–0.59)	<0.0001
No	107,516 (79.09)	46,342 (70.54)	1	
**Water service continuity (last 14 days)**				
Yes, Interrupted for a full day or more	57,016 (41.94)	28,879 (43.96)	1	
No, not interrupted for a full day	78,930 (58.06)	36,815 (56.04)	1.07 (1.05–1.09)	<0.0001
**Presence of water at hand washing stations**				
Water not available	90,471 (66.55)	9,082 (13.82)	0.07 (0.07–0.08)	<0.0001
Water is available	45,475 (33.45)	56,612 (86.18)	1	

The geographic location of residence, sub-region, community poverty, and community education were all found to be significantly associated with hand washing using essential agents on a community level (Table 9).

**Table 9:** Multilevel bivariate logistic regression analysis of factors associated with handwashing with essential agents, analysis from 18 countries, 2016–2021.

Variable categories	Hand washing with essential agents		COR (95% CI)	p-value
	No	Yes		
**Place of residence**				
Urban	39.546 (29.09)	35,843 (54.56)	2.99 (2.93–3.06)	<0.0001
Rural	96,400 (70.91)	29,851 (45.44)	1	
**Sub-Region**				
West Africa	53,207 (39.14)	28,299 (43.08)	0.44 (0.42–0.46)	< 0.0001
Middle Africa	7,758 (5.71)	9,067 (13.8)	1.26 (1.19–1.33)	< 0.0001
Eastern Africa	70,045 (51.52)	23,603 (35.93)	0.33 (0.31–0.34)	< 0.0001
Southern Africa	4,936 (3.63)	4,725 (7.19)	1	
**Community Poverty**				
Low	72,649(53.44)	28,103 (42.78)	1	
High	63,297 (46.56)	37,591 (57.22)	2.2 (2.00–2.48)	< 0.0001
**Community Education**				
Low	73,020 (53.71)	27,777 (42.28)	1	
High	62,926 (46.29)	37,917 (57.72)	2.10 (1.88–2.35)	< 0.0001

#### Factors associated with handwashing practice with essential agents multivariable multilevel mixed effects logistic regression analysis

After controlling for other variables, families with highly educated heads had a 77% higher likelihood of washing their hands with soap and water [aOR = 1.77; 95%(CI = 1.68–1.86)]. The sex of the household heads had a substantial impact on the likelihood of using essential agents for handwashing, with female-headed households being more likely to do so [aOR = 1.09; 95%(CI = 1.06–1.12)].

In comparison to households in the lowest quintile, households in the highest wealth quintile were four times more likely to follow hand washing recommendations[aOR = 4.08; 95%(CI = 3.84–4.33)]. A higher likelihood of utilizing essential agents was likewise associated with not using a shared toilet facility inside a household or compound [aOR = 1.13; 95%(CI = 1.10–1.17)]. Regarding water supply, not having regular access to water at hand washing stations and households with a fixed place for hand washing were both significantly associated with handwashing with essential agents [aOR = 0.09; 95%(CI = 0.095–0.10)]and [aOR = 1.49; 95%(CI = 1.45–1.54)]. Rural residents were 15% less likely to follow handwashing recommendations than urban residents [aOR = 0.85; 95%(CI = 0.82–0.88)] (Table 10).

**Table 10:** Multilevel multivariable logistic regression analysis of the factors associated with handwashing with essential agents, analysis from 18 countries, 2016–2021.

Variable categories	Null Model	Model-I (AOR) (95% CI)	Model-II (AOR) (95% CI)	Model-III (AOR) (95% CI)
**Age of household head**				
<18		1		1
19–30		1.08 (0.90–1.29)		1.09 (0.91–1.31)
31–45		1.16 (0.98–1.39)		1.16 (0.97–1.39)
46–65		1.29 (1.08–1.54)*		1.27 (0.96–1.52)
>66		1.23 (1.03–1.47)*		1.21(0.91–1.45)
**Educational status of HH head**				
No formal education		1		1
Primary education		1.14 (1.10–1.18)*		1.25 (1.20–1.30)*
Secondary education		1.47 (1.42–1.53)*		1.40 (1.34–1.45)*
Higher education		1.84 (1.75–1.93)*		1.77 (1.68–1.86)*
**Sex of HH head**				
Male		1		1
Female		1.10 (1.06–1.13)*		1.09 (1.06–1.12)*
**Household wealth**				
Poorest		1		1
Poorer		1.5 (1.45–1.61)*		1.46 (1.38–1.54)*
Middle		1.93 (1.83–2.03)*		1.81 (1.71–1.90)*
Richer		2.63 (2.49–2.77)*		2.5 (2.37–2.64)*
Richest		3.96 (3.75–4.19)*		4.08 (3.84–4.33)*
**Location of water source**				
On-premises		1.11 (1.08–1.15)*		1.02 (0.98–1.05)
Off Premises		1		1
**Share toilet with other home/s**				
Yes		1		1
No		1.12 (1.09–1.15)*		1.13 (1.10–1.17)*
**The place where members wash their hands**				
Fixed place		1.48 (1.44–1.52)*		1.49 (1.45–1.54)*
Mobile place		1		1
**Effective water treatment**				
Yes		1		1
No		0.72(0.70–0.75)*		0.64 (0.62–1.06)
**Presence of water at hand washing stations**				
Water not available		0.09 (0.09–0.095)*		0.09 (0.095–0.10)*
Water is available		1		1
**Place of residence**				
Urban			1	1
Rural			0.388 (0.38–0.397)*	0.85 (0.82–0.88)*
**Community Education**				
Low			1	1
High			1.45 (1.29–1,63)*	1.18 (0.97–1.29)

Key: 1: Reference category, AOR = Adjusted odds ratio, COR = Crude odds ratio,

* statistically significant at p-value <0.05

## Discussion

Poverty is perpetuated by a lack of access to proper hygiene. For both people and society as a whole, having access to running water and soap reduces obstacles to economic development. It could result in fewer lost workdays, lower healthcare expenses, and greater access to educational opportunities [16]. The present study assessed the prevalence of handwashing using essential agents in 18 countries via data from the Demographic and Health Survey. Of the assessed households, only 34.84% had access to essential amenities where family members could wash their hands. This result was consistent with the global average, according to which 40% of individuals worldwide, or 3 billion people, lack access to a facility for washing their hands with water and soap at home [29]. This means that millions of people are still unable to wash their hands with soap where they are born, raised, and educated and the simplest means of self- and family protection are inaccessible to the most vulnerable communities.

A disease must be seen as a significant community public health concern to be managed sustainably [21]. Previous research has shown a correlation between reduced handwashing rates and a lack of formal education [5, 15]. Likewise, in the current study, households with highly educated heads were also more likely to wash their hands with soap and water. This demonstrates the need to target households with messages that are developed using evidence from such findings to encourage acknowledgment of the universal susceptibility to WASH-related diseases.

In this study, households in the highest wealth quintile had a fourfold higher likelihood of washing their hands as per recommended standards. It is well known that acquiring and cleaning water, as well as the cost of soap, consume a sizable portion of a poor family’s resources [30]. In the context of widespread poverty, households’ inability to acquire resources may be the only thing preventing them from continuing the practice. Estimates show that the average economic return on investment for every dollar invested in water and sanitation is $4 [31]. Thus, increased domestic resource mobilization and donor-funded official development assistance flows are needed to make capital expenditures to address WASH infrastructure deficits.

In Africa, female-headed homes are typically poorer than male-headed homes, yet research also demonstrates that the children from these homes fare much better [32]. Children in homes with female heads score significantly better on long-term nutritional status indicators. Studies show that women are much more efficient than men in investing more in their families’ well-being [33, 34]. In the current study, households with female heads were more likely to wash their hands with essential agents. Therefore, it should be a priority in future projects to include women as partners, experts, and agents of change.

Not sharing toilets with other homes was positively associated with washing hands with soap and water. The refusal of homes to share essential handwashing supplies with other households and toilet users is a significant contributor to the lack of water and/or soap in shared spaces. This act is a reaction to the "free riding" concern, which arises when it is believed that individuals profit from something without contributing to its provision [17]. Future intervention models should therefore prioritize community involvement in their WASH programs and link communities to other stakeholders through coordination and advocacy mechanisms to ensure that those who manage their sanitation needs in shared or public spaces have a voice in the formulation of WASH service delivery plans and their implementation.

In the current study, contextual factors like regular access to water and the technology’s physical placement were significant predictors of handwashing with essential agents. A rising corpus of research, backed by behavioural plausibility, shows that if the aim is to promote habit formation, an interventional method that is much more automatic, unconscious, and cue-driven needs to be given more consideration [35]. For example, a handwashing station with water at a location designated for the action, like next to a toilet or in the kitchen, may encourage the behavior [36]. Likewise, continuous access to water serves as an important behavioural cue for handwashing practice and generally improves health outcomes [30]. Moving forward, the physical characteristics of the handwashing station are essential to understanding and promoting handwashing with soap since some products encourage the activity. Furthermore, more funding needs to be invested in capital projects to make up for deficiencies in the WASH infrastructure.

In the current study, urbanicity and handwashing with essential agents were associated. 57% of the water supply in sub-Saharan Africa’s rural households is unimproved [37]. Little is known about how and why some rural households improve their handwashing practices while others do not. Deficiencies, uneven service delivery, inequity, and inefficiency are acknowledged to be some of the major problems [38]. Thus, as part of their program, local governments should engage in extensive outreach and distribution to increase the number of establishments selling soap and other essentials, while also increasing investment to address the urban-rural divide.

### Limitations and strengths

Using nationally representative, population-level, multi-country data, this study is one of the first of its kind to examine the relationships between domestic environmental conditions and handwashing with essential agents. The relatively high sample sizes that were examined in this study are one of its strengths since they improve the accuracy of the results. Additionally, this study serves as a resource for program managers and policymakers that are implementing multi-sectoral planning to scale up hand-washing practices. However this study is not without limitations, similar to any cross-sectional study, associations can be established but not causal relationships. Because the family head of household frequently responds to questions about water and sanitation, this study is limited by its reliance on him or her. The head of the home could be a man or woman from one of the various age groups found in the households. As a result, there may be variations in the reporting of WASH questions depending on who completes the household questionnaire.

### Implications for policy and research

For many people, washing their hands with soap and water is more or less a fluid practice. Being aware of such variation in practice may be especially helpful for program developers when preparing for potential interventions and evaluation studies. To ensure that different types of users are taken into account and that ongoing training messages are tailored to each user, program designers should be aware of the various categories that households may fall into (e.g., unfaltering practitioners, resource-dependent practitioners, non-practitioners, etc.). Further, examining sub-national data for countries would be important for all of the countries to design effective and focused interventions.

### Conclusion and recommendation

In terms of showcasing improvements in handwashing practices, sub-Saharan countries are off-course. There are still many nations without access to household water sources and fundamental sanitation infrastructure. The implementation of WASH initiatives will be crucial for the success of essential agent adoption programs in resource-limited settings. This includes educating the public on how to put up basic handwashing stations using affordable, locally accessible materials. Additionally, beyond everyday routines, the social context as well as the wider environment may have an impact on continuous handwashing behavior. Therefore, it is crucial to understand the factors that discourage people from using essential agents, including socio-cultural and psychosocial ones like educational status, female headship, household wealth, not using a shared toilet facility as well as contextual factors like regular access to water, handwashing technology’s physical placement, and urbanicity.

## Abbreviations

AIC: Akaike’s information criterion
AOR: Adjusted odds ratio
BIC: Schwarz’s Bayesian information criteria
COR: Crude odds ratio
DHS: Demographic and health survey
HR: Household data
ICC: Intra Class Correlation Coefficient
MOR: Median Odds Ratio
PCV: Proportional Change in Variance
VIF: Variance inflation factor
WASH: water, sanitation, and hygiene
